# Association between The Number of Retrieved Mature Oocytes and Insulin Resistance or Sensitivity in Infertile Women Polycystic Ovary Syndrome

**DOI:** 10.22074/ijfs.2019.5422

**Published:** 2018-10-02

**Authors:** Fatemeh Hassani, Shahrbanoo Oryan, Poopak Eftekhari-Yazdi, Masood Bazrgar, Ashraf Moini, Nahid Nasiri, Azadeh Ghaheri

**Affiliations:** 1 Department of Animal Biology, Faculty of Biological Sciences, Kharazmi University, Tehran, Iran; 2 Department of Embryology, Reproductive Biomedicine Research Center, Royan Institute for Reproductive Biomedicine, ACECR, Tehran, Iran; 3 Department of Genetics, Reproductive Biomedicine Research Center, Royan Institute for Reproductive Biomedicine, ACECR, Tehran, Iran; 4 Department of Endocrinology and Female Infertility, Reproductive Biomedicine Research Center, Royan Institute for Reproductive Biomedicine, ACECR, Tehran, Iran; 5 Department of Obstetrics and Gynecology, Arash Women’s Hospital, Tehran University of Medical Sciences, Tehran, Iran; 6 Department of Epidemiology and Reproductive Health, Reproductive Epidemiology Research Center, Royan Institute for Reproductive Biomedicine, ACECR, Tehran, Iran

**Keywords:** Assisted Reproductive Technology, Insulin Resistance, Oocyte, Polycystic Ovary Syndrome

## Abstract

**Background:**

The objective of this study was to describe the association between luteinizing hormone (LH)/ follicle-stimulating hormone (FSH) ratio and demographic variables and maturation stage of oocytes in insulin- resistant and insulin-sensitive patients with polycystic ovary syndrome (PCOS) in comparison with control group.

**Materials and Methods:**

In this case-control study, 60 patients with *in vitro* fertilization (IVF)/intracytoplas- mic sperm injection (ICSI) indication were subdivided into 3 groups as follow: 20 subjects were assigned to control (fertile women with male infertility history) group, 20 subjects with PCOS were insulin resistant (IR) and 20 subjects with PCOS were insulin sensitive (IS). After puncture, retrieved oocytes were classified into metaphase II (MII) as mature and in metaphase I (MI) or germinal vesicle stage (GV) as immature. Regres- sion analyses were used to explore the association between MII oocyte number and demographic and clinical variables.

**Results:**

LH/FSH ratio was significantly higher in PCOS-IR women compared to controls but not significantly dif- ferent from that of PCOS-IS group. PCOS-IR women had lower MII oocyte number compared with that of controls. According to multiple regression analysis, the number of previous assisted reproductive technology (ART) cycles was negatively associated with the number of MII oocytes.

**Conclusion:**

Insulin resistance can be associated with reductions in MII oocyte number in patients with PCOS.

## Introduction

Polycystic ovary syndrome (PCOS) is a common endocrine disorder in women of reproductive age, which is mostly associated with hyperinsulinemia, hyperandrogenism and anovulatory infertility (1, 2). The prevalence of PCOS is 6-26% depending on differences in study populations background and the applied diagnostic criteria (3, 4).

Follicular arrest (FA) and dysregulation of paracrine activity in follicles are noticeable ovarian signs in PCOS patients (5). Insulin plays an important role in regulating the response of human follicular cells to gonadotropins (6). Evaluation of the association between insulin resistance and ovarian hyperstimulation syndrome (OHSS) revealed that hyperinsulinemia may lead to disruption of ovarian steroidogenesis, which in turn increases the secretion of ovarian androgens by dysregulation of cytochrome P450c17α activity (7, 8). High levels of insulin can occupy the insulin-like growth factor-1 (IGF-1) receptors therefore simulating and disturbing their function and subsequently resulting in hyperandrogenism (9).

Recently, it has been observed that insulin can modulate steroidogenesis through its own receptor. Moreover, in case of insulin resistance, the steroidogenesis appears to be preserved likely by various mechanisms of regulation of receptors receptivity in different tissue (10). *In vitro* studies using cultured pituitary cells have demonstrated that insulin increases the luteinizing hormone (LH) secretion during the enhancement of pituitary responsiveness to gonadotropin releasing hormone (GnRH) (11, 12). Furthermore, DiVall et al. (13) observed that increased insulin receptor signaling in GnRH neurons of obese female mice, elevated GnRH pulsatile secretion and consequent LH secretion resulting in reproductive abnormality.

Excessive ovarian androgen production has also been implicated in the pathogenesis of PCOS. It has been postulated that hyperinsulinemia in case of insulin resistance, is associated with capacity of ovarian androgen production (4, 5). Severe insulin resistance causes a compensatory hyperinsulinemia, which stimulates ovarian androgen production in the presence of sufficient LH (14).

An *in vitro* study showed that insulin and IGF-I stimulate androgen production in incubated human stroma and theca cells. In some women with insulin-resistance-induced hyperandrogenism, an acute rise in circulating androgens may be induced by increases in glucose concentration. The effect of circulating androgen rise is dependent on the amount of insulin secreted in response to glucose enhancement. These data suggest that hyperinsulinemia may play a central role in the development of ovarian hyperandrogenism (14). According to a previous study, insulin can induce steroid secretion (i.e. insulin acts as a co-gonadotropin) (15). Increased LH serum level and enhanced ratio of LH/follicle-stimulating hormone (FSH) are seen in many of PCOS patients. Frequent coexistence of elevated LH and increased insulin concentrations leads to more severe manifestations of PCOS manifestations (15, 16).

Folliculogenesis and oocyte maturation are complex processes that require the action of both LH and FSH (17, 18). In PCOS patients high LH levels have been associated with significant decreases in oocyte maturation and fertilization rates, and impaired embryo quality (17, 19). Hyperinsulinemia may impair the competence of oocyte development. Subsequently, higher percentages of low-quality oocytes in PCOS may cause lower fertilization rates and decreased embryos quality that have been reported in PCOS patients compared to healthy women with assisted reproductive technology (ART) indication (18, 19).

In this study, we prospectively evaluated the association between different factors [age, body mass index (BMI), number of previous ART cycle, and LH/FSH ratio] and oocyte maturity in insulin-resistant and insulin-sensitive women with PCOS in comparison with control group.

## Materials and Methods

### Patient selection

In this case-control study, each of 40 patient’s case seeking assisted reproduction at Royan Institute from April 2014 to January 2015 was analyzed. Written informed consent was obtained from all the participants. Ethics approval was obtained from the local Ethics committee of Royan Institute (no.EC/93/1138). PCOS patients were allocated to one of the two groups formed based on the level of fasting insulin (FI): insulin resistant (PCOS-IR; FI≥12 mg/dl) and insulin sensitive (PCOS-IS; FI<12 mg/dl). In control group, 20 women with regular menstrual cycle without known diseases (i.e. fertile women with male infertility history) were included. Exclusion criteria were impaired thyroid, renal or hepatic function, congenital adrenal hyperplasia (CAH), endometriosis, premature ovarian insufficiency (POI), functional hypothalamic amenorrhea (FHA), unexplained infertility (UI) and age>36 years.

### Stimulation protocol

In order to controlled induce ovarian stimulation (COS), daily subcutaneous injection of recombinant human FSH (rFSH, Gonal F®; Serono Pharma, Switzerland) was started from the second day of the cycle. Starting dose of rFSH was adjusted individually depending on patients response measured by transvaginal ultrasonography, antral follicle count (AFC), levels of serum estradiol (E2) and AMH. A GnRH antagonist-cetrorelix (Cetrotide®, Merck Serono, Germany) was administered subcutaneously when at least two ovarian follicles reached 14 mm in diameter. The protocol consisted of daily subcutaneous injections of Cetrotide 0.25 mg, until the criteria for human chorionic gonadotropin (hCG) administration were met. For final oocyte maturation, when the dominant follicle reached ≥18 mm in diameter with the following two follicles ≥16 mm and E2 levels between 1000-4000 pg/mL, an intramuscular injection of 10.000 IU hCG (Pregnyl®, Organon, Holland) or subcutaneous injection of 250 μg hCG (Ovitrelle®, Merck Serono, France) was given.

### Oocytes retrieval

Oocyte Pick-up (OPU) was done using transvaginal ultrasound-guided follicle aspiration, 36 hours after hCG administration to collection tubes. Following OPU, cumulus-oocyte complexes were washed several times in fertilization medium (G-IVF®, Vitrolife, Sweden) to remove blood and cell debris, and incubated for two hours in fertilization medium (G-IVF®, Vitrolife, Sweden). Retrieved oocytes were classified into metaphase II (MII) stage as mature and metaphase I (MI) or germinal vesicle (GV) stage as immature. Oocyte denudation was performed using 80 IU of hyaluronidase (Sigma, USA) (20). Participants underwent intracytoplasmic sperm injection (ICSI). The spermatozoa were prepared using density gradient centrifugation (AllGrad®; LifeGlobal, USA) for PCOS patients or standard swim-up method for control group. PCOS patients' quality of sperm were compatible with WHO criteria 2010; however, in the control group, male factor infertility existed with respect to sperm quality (i.e. oligo, asteno, teratozoospermia or combinations of these conditions) according to the WHO parameters. After sperm microinjection into the MII oocytes, fertilization was confirmed 16 to 17 hours after ICSI, by the presence of two pronuclei (2PN) and a second polar body. Zygotes were individually placed in 20 μl fresh G-1TM medium (Vitrolife) supplemented with 10% recombinant human serum albumin (HAS-solutionTM, Vitrolife) under oil (OVOILTM, Vitrolife) for a 72 hour culture.

### Embryological assessment

Based on our laboratory standards, embryos were graded at the pronuclear and cleavage stages. The quality of the embryos at cleavage stage were classified according to the following criteria: [excellent quality (≥4 cells or ≥8 cells and <10 % fragmentation), good quality (≥ 4 cells or ≥8 cells and 10-20% fragmentation) and poor quality (<4 cells or <8 cells and >20 % fragmentation)] (21, 22). Decision on the number of transferred embryos was made based on 2013 ASRM embryo-transfer guidelines (23). Seventy two hours after ICSI, mainly a maximum of two embryos of excellent grade or good quality were transferred to uterine cavity by a Labotect catheter (Labotect, Germany).

### Luteal support

On the day of oocyte retrieval, luteal phase support included Cyclogest® 200 mg (Actavis, UK) vaginal suppositories, twice daily (bid) for 14 days. Endometrial thickness was between 8 to 11 mm and showed a triple-line pattern as examined by vaginal ultrasonography on the day of hCG injection. Gestation was confirmed by pregnancy test 14 days after ET. Clinical pregnancy confirmed when a gestational sac with fetal cardiac activity was detectable after 7 weeks of gestation. Biochemical gestation was not taken into consideration at any stage of the study.

### Statistical analysis

In this study, categorical variables are presented as number (%) and continuous variables as mean ± SD or median (minimum-maximum; inter-quartile range) where appropriate. Statistical comparisons of means of the three study groups were performed using ANOVA or its nonparametric equivalent, Kruskal-Wallis test. Independent t-test was used to assess mean differences in FI between IR and IS- PCOS groups. Chi-square analysis was used for qualitative data. Univariate and backward multiple linear regression, including all variables, were used to evaluate the association between MII oocyte number and some demographic and clinical variables. Statistical analyses were performed using IBM SPSS Statistics for Windows, Version 22.0 (IBM Crop., Armonk, NY, USA). All statistical tests were 2-tailed and a P<0.05 was considered statistically significant.

## Results

Clinical characteristics of participants are shown in Table 1. BMI was significantly higher in PCOS-IR group (29.97 ± 4.39) compared to control group (25.12 ± 4.21 P=0.023), this difference was not significant between PCOS-IS (26.31 ± 8.03) and either of PCOS-IR and control groups. PCOS-IR women had significantly fewer number of MII oocytes (8.10 ± 3.61) compared to controls (11.57 ± 5.11, P=0.028); but, the number of MII oocytes was not significantly different between PCOS-IS women (9.05 ± 3.37) and control subjects. There were no significant differences in MI, GV and dead oocytes between PCOS groups (IR and IS) and control group. Fasting insulin was significantly higher in PCOS-IR (19.30 ± 9.60 mg/dl) compared to PCOS-IS group (6.67 ± 2.89 mg/dl, P=0.006). Other criteria including age, previous ART history, number of retrieved oocytes, number of 2PN embryos, total number of embryo and success rate did not differ significantly among the three groups.

**Fig.1:**
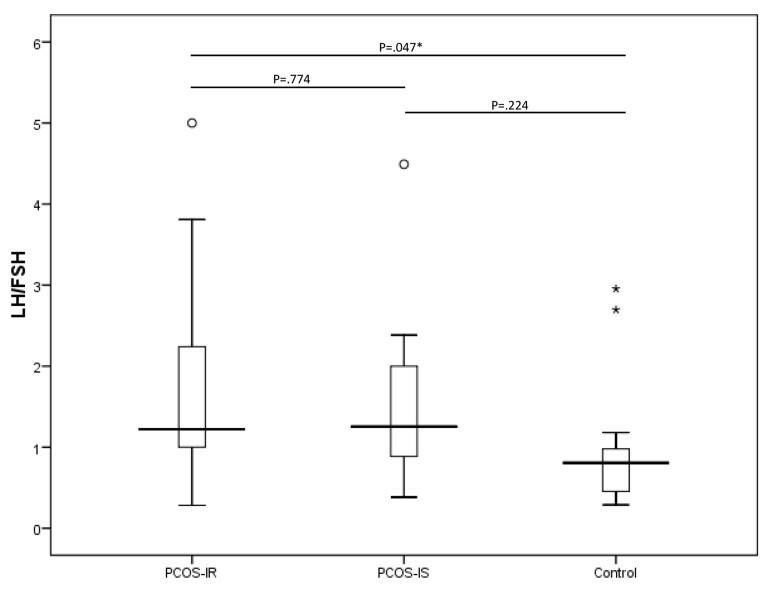
Boxplots of LH/FSH in PCOS-IR, PCOS-IS and control group. LH; Luteinizing hormone, FSH; Follicle-stimulating hormone, PCOS; Polycystic ovary syndrome, IR; Insulin resistant, and IS; Insulin sensitive. Extreme values are indicated using asterisks and outliers are indicated using circles.

LH/FSH ratio was significantly higher in PCOS-IR women (1.67 ± 1.75) compared to controls (0.94 ± 0.68, P=0.047) but not significantly different from that of PCOS-IS group (1.45 ± 0.94) (Fig .1). Regression analysis results are presented in Table 2. On the basis of the univariate analysis, LH/FSH in study groups (PCOS-IR, PCOS-IS and control) were significantly associated with MII oocyte number. After adjusting for other variables, based on the multiple linear regression model results, LH/FSH was no longer statistically significant; however, on average, the PCOS-IR group had 4.09 MII oocytes less than the control group and the PCOS-IS group had 3.21 MII oocytes less than the control group. Multiple linear regression model also identified that the number of previous ART cycles was negatively associated with MII oocyte number. As shown in Table 2, for each unit increase in previous ART cycle number, the expected number of MII oocyte decreases by 1.23. Other variables included in the univariate model and displayed in Table 2, were not significantly associated with MII oocyte number in the multiple model.

**Table 1:** Demographic and clinical characteristics of recruited patients


	PCOS-IR n=20	PCOS-IS n=20	Control n=20	P value

Age (Y)	30.04 ± 4.45	28.00 ± 4.00	29.05 ± 5.18	0.381
Previous ART				
Yes	7 (25.9)	7 (25.9)	13 (48.2)	0.108
No	15 (42.9)	12 (34.3)	8 (22.8)	
Fasting insulin (mg/dl)	19.30 ± 9.60	6.67 ± 2.89	-	0.006
BMI	29.97 ± 4.39^a^	26.31 ± 8.03^a^^b^	25.12 ± 4.21^b^	0.023
Oocyte retrievedMIIMIGVDegenerated/dead	11.90 ± 7.098.01 ± 3.61^a^0 (0-3; 1)0 (0-2; 0)0 (0-1; 0)	13.58 ± 11.269.05 ± 3.37^a^^b^0 (0-2; 0)0 (0-4; 1)0 (0-4; 1)	12.90 ± 5.59111.57 ± 5.11^b^0 (0-1; 1)0 (0-3; 1)0 (0-2; 0.5)	0.8140.0280.8060.4680.125
Total embryo	7.15 ± 4.29	9.44 ± 7.34	7.95 ± 4.07	0.415
2PN embryos	6 (0-14; 4.75)	7 (1-13; 3.25)	9 (2-18; 4.5)	0.723
Fertilization (%)	73.28 ± 25.43	75.90 ± 25.89	76.01 ± 17.24	0.914


PCOS; Polycystic ovary syndrome, IR; Insulin resistant, IS; Insulin sensitive, ART; Assisted reproductive technology, BMI; Body mass index, MI; Metaphase I oocytes, MII; Metaphase II oocytes, GV; Germinal vesicle stage, 2PN; Two pronuclei. Continuous variables are presented as mean ± SD or median (minimum-maximum; inter-quartile range) when appropriate. Categorical variables are presented as number (%). Different letters indicate significantly different means (as evaluated by ANOVA and Tukey post-hoc test). A P<0.05 was considered significant.

**Table 2:** Regression analysis for factors associated with MII oocytes number


	Univariate analysis			Multiple analysis		
	B	SE	P value	B	SE	P value

Age	-0.01	0.13	0.910			
BMI	-0.14	0.09	0.143			
LH/FSH	-1.22	0.56	0.035			
No. of previous ART cycle	-0.71	0.61	0.251	-1.23	0.59	0.043
Group						
PCOS-IR vs. control	-3.47	1.30	0.010	-4.09	1.29	0.003
PCOS-IS vs. control	-2.52	1.33	0.064	-3.21	1.34	0.020


B; Unstandardized coefficient, SE; Standard error, BMI; Body mass index, LH; Luteinizing hormone, FSH; Follicle-stimulating hormone, ART; Assisted reproductive technology, PCOS; Polycystic ovary syndrome, IS; Insulin sensitive, and IR; Insulin resistant.

## Discussion

PCOS is a heterogeneous endocrinopathy; insulin resistance and elevated LH/FSH ratio play a potential role in the pathogenesis of the disorder (24). However, according to the 2003 rotterdam ESHRE/ASRM-sponsored PCOS Consensus workshop group, increased LH/FSH ratio and IR will not be considered the main criteria for the diagnosis of PCOS and more research is needed in this area (19). Banaszewska et al. (25) studied a rare subgroup of PCOS women and observed that increased LH levels and IR occurred simultaneously. In this study, in terms of biochemical factors, there was a significant increase in LH/FSH ratio in PCOS-IR compared to control group (P<0.05). We described the demographic characteristic of these patients and the outcome of IVF/ICSI treatment. According to our data, in the PCOS-IR group, mean number of MII was almost 4 units less than that of the control group. Moreover, in the PCOS-IS group, the number of mature oocytes was on average almost 3 units lower than that of the healthy women. As shown by Colton et al. (26), continuation of meiosis and maturation of oocytes were defected in a diabetic mouse model. Also, it was reported that insulin resistance can impair normal fertilization or chromosomal abnormalities in affected oocytes (27). These findings explain aberrant relationships between oocytes and surrounding cumulus cells (26).

According to recent studies, insulin signals relay through multiple pathways, many of them are active in ovarian follicular cells especially oocytes. These pathways are able to interact with each other and also with gonadotropins. Thus, insulin has a direct regulatory effect on ovarian physiology (28). According to the results of Burghen et al. (29), insulin resistance was common in PCOS women even if the patients were obese. It was indicated that high levels of insulin can bind to not only insulin receptors but also ovarian IGF1 receptors, the latter stimulate steroidogenesis. In particular, this effect is mediated by aromatase activity (30). There is a synergism between insulin and gonadotropins (LH and FSH) that can intensify steroidogenesis. Poretsky and Kalin (30) have proposed that LH can induce interstitial cells of ovary to differentiate into androgen-producing cells. Hyperinsulinemia possibly accelerates this process. This hyperandrogenic condition would result in follicular atresia. Tarlatzis et al. (31) found that elevated local androgens and loss of estrogen production lead to follicular growth arrest.

The strengths of this study were evaluation of the effects of increased LH/FSH level and insulin resistance on the oocyte maturation. This study has a prospective scheme and all patients underwent ICSI and received GnRH antagonist. Besides, the statistical calculations were accomplished by multivariable analysis. Finally, we observed lower number of MII oocytes in IR patients. The mechanisms underlying these results are unclear; although previous studies showed that ovarian folliculogenesis may disrupted by high LH levels (32). Tarlatzis et al. (31) reported that elevated LH/FSH in human menopausal gonadotropin (HMG)-stimulated PCOS women may have a detrimental effect on the maturation of oocytes. However, according to Wiser et al. (33) higher numbers of mature oocytes were retrieved from PCOS women with higher LH/FSH ratio. Since according to the inclusion criteria of the study, only women who had indication for *in vitro* maturation (IVM) treatment were recruited, the results were inconsistent.

According to some studies, insulin resistance is not a disease but it can develop numerous metabolic alterations (34, 35). These metabolic disturbances can make lesions in cumulus- oocyte complexes (COCs) of murine. In fact, outbreak of insulin resistance may halt the progression of meiosis and postpone oocytes maturation (26). Although increased secretion of LH and insulin resistance is common characteristics of PCOS, in lean PCOS amplified growth hormone (GH) pulsatility in addition to LH hypersecretion induce theca cells to release androgens. In obese PCOS patients, elevated IGFs levels induced by insulin, stimulate granulosa cells to produce androgens. These processes enhance hyperandrogenemia and anovulation (36). Our data suggested that insulin resistance may diminish oocyte maturity.

According to previous studies, the success probability is essentially constant across the first three IVF attempts (37). In the other words, a maximum of three IVF treatment cycles lead to one child birth (38). Tan et al. (37) reported that there were no statistically significant differences in the pregnancy failure rates between those attempting a second course of treatment and those attempting the first course. In both groups of patients, the pregnancy failure rates were more or less the same in all age groups until the women were at least 35 years old (37, 39).

Based on the results of this study, for each unit increase in previous IVF/ICSI failure, the expected number of MII oocytes decreases by 1.23 in PCOS women. Moreover, to the best of our knowledge, the present study is the first report showing that the history of IVF/ICSI failure is associated with reduction of MII oocytes in PCOS-IR patients. Also, our results showed that retrieval of lower numbers of mature oocytes from PCOS women might be in part related to insulin resistance. Our study suggested that treatment of insulin resistance should be considered in PCOS-IR patients who have a history of canceled IVF cycles. This can help to achieve greater numbers of mature oocytes.

## Conclusion

Insulin resistance is a common metabolic abnormality in PCOS patients and PCOS-IR women had lower MII oocytes than control group. Collectively, histories of ART failure and insulin resistance are two important factors in predicting the number of mature oocytes in PCOS-IR patients.

## References

[1] Shah D, Rasool S (2016). Polycystic ovary syndrome and metabolic syndrome: the worrisome twosome?. Climacteric.

[2] Moghetti P, Tosi F, Bonin C, Di Sarra D, Fiers T, Kaufman JM (2013). Divergences in insulin resistance between the different phenotypes of the polycystic ovary syndrome. J Clin Endocrinol Metab.

[3] Tehrani FR, Simbar M, Tohidi M, Hosseinpanah F, Azizi F (2011). The prevalence of polycystic ovary syndrome in a community sample of Iranian population: Iranian PCOS prevalence study. Reprod Biol Endocrinol.

[4] Yildiz BO, Bozdag G, Yapici Z, Esinler I, Yarali H (2012). Prevalence, phenotype and cardiometabolic risk of polycystic ovary syndrome under different diagnostic criteria. Hum Reprod.

[5] Xi W, Yang Y, Mao H, Zhao X, Liu M, Fu S (2016). Circulating anti-mullerian hormone as predictor of ovarian response to clomiphene citrate in women with polycystic ovary syndrome. J Ovarian Res.

[6] Stocco DM, Wang X, Jo Y, Manna PR (2005). Multiple signaling pathways regulating steroidogenesis and steroidogenic acute regulatory protein expression: more complicated than we thought. Mol Endocrinol.

[7] Hayes MG, Urbanek M, Ehrmann DA, Armstrong LL, Lee JY, Sisk R (2015). Genome-wide association of polycystic ovary syndrome implicates alterations in gonadotropin secretion in European ancestry populations. Nat Commun.

[8] Unlühizarci K, Keleştimur F, Bayram F, Sahin Y, Tutuş A (1999). The effects of metformin on insulin resistance and ovarian steroidogenesis in women with polycystic ovary syndrome. Clin Endocrinol (Oxf).

[9] Homburg R, Pariente C, Lunenfeld B, Jacobs HS (1992). The role of insulin-like growth factor-1 (IGF-1) and IGF binding protein-1 (IGHassani FBP-1) in the pathogenesis of polycystic ovary syndrome. Hum Reprod.

[10] Barbieri RL (2008). Update in female reproduction: a life-cycle approach. J Clin Endocrinol Metab.

[11] Adashi EY, Hsueh AJ, Yen SS (1981). Insulin enhancement of luteinizing hormone and follicle-stimulating hormone release by cultured pituitary cells. Endocrinology.

[12] Allon MA, Leach RE, Dunbar J, Diamond MP (2005). Effects of chronic hyperandrogenism and/or administered central nervous system insulin on ovarian manifestation and gonadotropin and steroid secretion. Fertil Steril.

[13] DiVall SA, Herrera D, Sklar B, Wu S, Wondisford F, Radovick S (2015). Insulin receptor signaling in the GnRH neuron plays a role in the abnormal GnRH pulsatility of obese female mice. PLoS One.

[14] Barbieri RL, Smith S, Ryan KJ (1988). The role of hyperinsulinemia in the pathogenesis of ovarian hyperandrogenism. Fertil Steril.

[15] Codner E, Iñíguez G, Villarroel C, Lopez P, Soto N, Sir-Petermann T (2007). Hormonal profile in women with polycystic ovarian syndrome with or without type 1 diabetes mellitus. J Clin Endocrinol Metab.

[16] Allahbadia GN, Merchant R (2011). Polycystic ovary syndrome and impact on health. Middle East Fertil Soc J.

[17] Mermillod P, Oussaid B, Cognie Y (1999). Aspects of follicular and oocyte maturation that affect the developmental potential of embryos. J Reprod Fertil Suppl.

[18] Filicori M (1999). The role of luteinizing hormone in folliculogenesis and ovulation induction. Fertil Steril.

[19] Rotterdam ESHRE/ASRM-Sponsored PCOS Consensus Workshop Group (2004). Revised 2003 consensus on diagnostic criteria and long-term health risks related to polycystic ovary syndrome. Fertil Steril.

[20] Wissing ML, Sonne SB, Westergaard D, Nguyen Kd, Belling K, Host T (2014). The transcriptome of corona radiata cells from individual MІІ oocytes that after ICSI developed to embryos selected for transfer: PCOS women compared to healthy women. J Ovarian Res.

[21] Eftekhari-Yazdi P, Valojerdi MR, Ashtiani SK, Eslaminejad MB, Karimian L (2006). Effect of fragment removal on blastocyst formation and quality of human embryos. Reprod Biomed Online.

[22] Valojerdi MR, Karimian L, Yazdi PE, Gilani MA, Madani T, Baghestani AR (2006). Efficacy of a human embryo transfer medium: a prospective, randomized clinical trial study. J Assist Reprod Genet.

[23] Practice Committee of American Society for Reproductive Medicine, Practice Committee of Society for Assisted Reproductive Technology (2013). Criteria for number of embryos to transfer: a committee opinion. Fertil Steril.

[24] Moran C, Garcia-Hernandez E, Barahona E, Gonzalez S, Bermudez JA (2003). Relationship between insulin resistance and gonadotropin dissociation in obese and nonobese women with polycystic ovary syndrome. Fertil Steril.

[25] Banaszewska B, Spaczyński RZ, Pelesz M, Pawelczyk L (2003). Incidence of elevated LH/FSH ratio in polycystic ovary syndrome women with normo- and hyperinsulinemia. Rocz Akad Med Bialymst.

[26] Colton SA, Pieper GM, Downs SM (2002). Altered meiotic regulation in oocytes from diabetic mice. Biol Reprod.

[27] Diamond MP, Moley KH, Pellicer A, Vaughn WK, DeCherney AH (1989). Effects of streptozotocin- and alloxan-induced diabetes mellitus on mouse follicular and early embryo development. J Reprod Fertil.

[28] Dupont J, Scaramuzzi RJ (2016). Insulin signalling and glucose transport in the ovary and ovarian function during the ovarian cycle. Biochem J.

[29] Burghen GA, Givens JR, Kitabchi AE (1980). Correlation of hyperandrogenism with hyperinsulinism in polycystic ovarian disease. J Clin Endocrinol Metab.

[30] Poretsky L, Kalin MF (1987). The gonadotropic function of insulin. Endocr Rev.

[31] Tarlatzis BC, Grimbizis G, Pournaropoulos F, Bontis J, Lagos S, Spanos E (1995). The prognostic value of basal luteinizing hormone: follicle-stimulating hormone ratio in the treatment of patients with polycystic ovarian syndrome by assisted reproduction techniques. Hum Reprod.

[32] van der Spuy ZM, Dyer SJ (2004). The pathogenesis of infertility and early pregnancy loss in polycystic ovary syndrome. Best Pract Res Clin Obstet Gynaecol.

[33] Wiser A, Shehata F, Holzer H, Hyman JH, Shalom-Paz E, Son WY (2013). Effect of high LH/FSH ratio on women with polycystic ovary syndrome undergoing in vitro maturation treatment. J Reprod Med.

[34] Reaven GM (2005). The insulin resistance syndrome: definition and dietary approaches to treatment. Annu Rev Nutr.

[35] Diamanti-Kandarakis E, Dunaif A (2012). Insulin resistance and the polycystic ovary syndrome revisited: an update on mechanisms and implications. Endocr Rev.

[36] Morales AJ, Laughlin GA, Butzow T, Maheshwari H, Baumann G, Yen SS (1996). Insulin, somatotropic, and luteinizing hormone axes in lean and obese women with polycystic ovary syndrome: common and distinct features. J Clin Endocrinol Metab.

[37] Tan SL, Doyle P, Maconochie N, Edwards RG, Balen A, Bekir J (1994). Pregnancy and birth rates of live infants after in vitro fertilization in women with and without previous in vitro fertilization pregnancies: a study of eight thousand cycles at one center. Am J Obstet Gynecol.

[38] Olivius K, Friden B, Lundin K, Bergh C (2002). Cumulative probability of live birth after three in vitro fertilization/intracytoplasmic sperm injection cycles. Fertil Steril.

[39] Schmittlein DC, Morrison DG, Carrell DP, Peterson CM (2010). A live baby or your money back: the marketing of in vitro fertilization procedures. Reproductive endocrinology and infertility: integrating modern clinical and laboratory practice.

